# Favorable survival with combined treatment in a metastatic breast cancer patient undergoing hemodialysis: A case report

**DOI:** 10.1016/j.ijscr.2021.01.044

**Published:** 2021-01-15

**Authors:** Megumi Matsumoto, Hiroshi Yano, Ryota Otsubo, Aya Tanaka, Takeshi Nagayasu

**Affiliations:** Department of Surgical Oncology, Nagasaki University Graduate School of Biomedical Sciences, Japan

**Keywords:** HD, hemodialysis, CKD, chronic kidney disease, CT, computed tomography, PET/CT, positron emission tomography/computed tomography, VA, vascular access, CVport, central venous access port, RDI, relative dose intensity, T-mab, trastuzamab, P-mab, pertuzumab, FN, febrile neutropenia, T-DM1, trastuzumab emtansine, EC, epirubicin and cyclophosphamide, PTX, paclitaxel, VNR, vinorelbine, AE, adverse event, DTX, docetaxel, Metastatic breast cancer, Haemodialysis, Dosage adjustment, Vascular access

## Abstract

**Introduction:**

Management of breast cancer patients undergoing hemodialysis (HD) is difficult because of a lack of evidence about drug selection, dose adjustment, and surgical procedures. We herein present a case of metastatic breast cancer in a patient undergoing HD.

**Presentation of case:**

A 58-year-old Japanese woman with breast cancer undergoing HD underwent total mastectomy of the left breast and left axillary dissection. Histopathological examination revealed invasive ductal carcinoma, and the diagnosis was pT2N3cM0 Stage ⅢC. Immunostaining of the resected specimen indicated that the tumor was estrogen receptor-positive, progesterone receptor-negative, human epithelial growth factor receptor 2-positive, and the Ki-67 labeling index was 70%. A postoperative positron emission tomography/computed tomography (PET/CT) scan indicated fluorodeoxyglucose uptake in the supraclavicular nodes. She received adjuvant therapy of epirubicin and cyclophosphamide followed by docetaxel, trastuzumab (T-mab) and radiation therapy. However, she developed multiple liver metastases during adjuvant T-mab and hormone therapy. Therefore, her regimen was changed to trastuzumab emtansine (T-DM1) as first-line therapy, T-mab, pertuzumab (P-mab), and eribulin as second-line therapy, and T-mab, P-mab, and weekly paclitaxel as third-line therapy. Eventually, she was administered fourth-line treatment of T-mab, P-mab, and vinorelbine because of adverse events. She has survived more than 25 months after the initial detection of recurrence of breast cancer and maintained quality of life.

**Conclusion:**

We report a case of breast cancer in a patient undergoing HD. It is very difficult to identify the appropriate drugs and dosages in patients undergoing HD to improve survival and quality of life.

## Highlights

• Few studies have examined management of breast cancer patients on hemodialysis.
• Dosage for anticancer drugs should be carefully adjusted based on adverse events.
• Combined therapy improves survival and quality of life.

## 1 Introduction

The risk of cancer, including breast cancer, is increased among patients undergoing hemodialysis (HD) [1,2]. In the United States, women undergoing HD are 42% more likely to develop breast cancer than the general population [1]. Unlike age, family history of breast cancer, early menarche, late menopause, late pregnancy, long-term hormone replacement therapy, radiation exposure, and benign breast diseases, chronic kidney disease (CKD) is not a major risk factor for breast cancer [3,4].

There are few studies on anticancer drugs and other drugs used in cancer therapy in patients undergoing HD, even though CKD, HD, and peritoneal dialysis do not limit the possibility of surgical treatment and radiotherapy. We herein present a case of HD with relatively long-term survival after diagnosis of multiple liver metastases detected during adjuvant therapy after surgery for locally advanced breast cancer.

## 2 Presentation of case

**Fig. 1:**
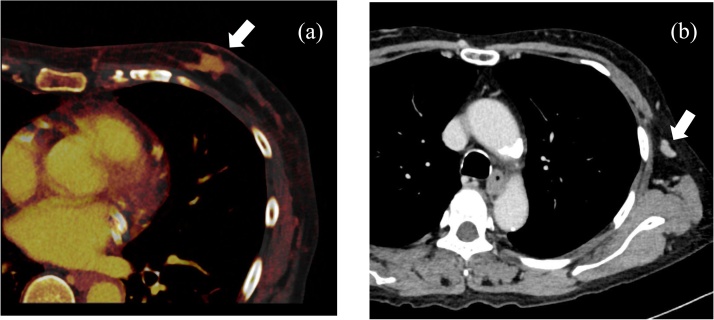
(a) Dual-source computed tomography (CT) revealed breast cancer, and (b) plane CT revealed metastatic axillary lymph nodes.

A 58-year-old Japanese woman undergoing HD developed a nodule in the left breast that was observed on chest computed tomography (CT). A dual-source CT scan showed an enhanced breast nodule 2.3 cm in diameter in the left breast (Fig. 1a), and enhanced CT indicated a swollen axillary lymph node (Fig. 1b). Needle biopsy was performed, and the pathological findings of the specimen indicated invasive ductal carcinoma that was estrogen receptor-positive, progesterone receptor-negative, and human epidermal growth factor receptor 2-positive by immunohistochemistry. Fine-needle aspiration cytology of the left axillary lymph node showed malignancy. The patient was diagnosed with primary breast cancer (cT2N1MX Stage ⅡB according to the Union for International Cancer Control classification).

**Fig. 2:**
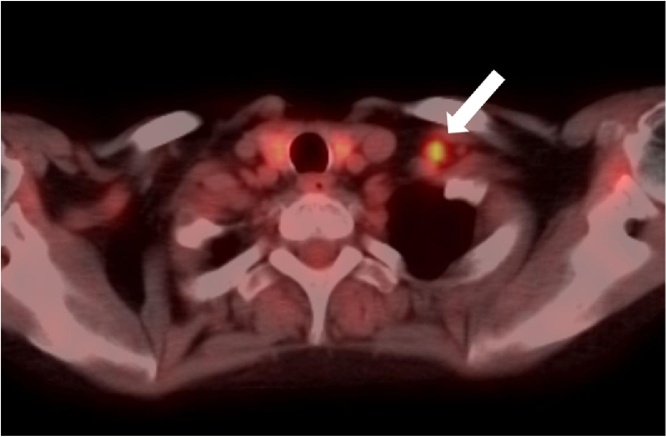
Positron emission tomography (PET)/computed tomography showed a hot spot in a metastatic supraclavicular lymph node (arrow).

She underwent total mastectomy of the left breast and left axillary dissection. The pathological evaluation confirmed invasive ductal carcinoma (2.4 cm) and 10 metastatic axillary lymph nodes. A postoperative positron emission tomography/computed tomography (PET/CT) scan showed uptake in the supraclavicular lymph nodes (Fig. 2) but not in distant organs (pT2N3cM0 Stage IIIC).

We administered the combined systemic therapy regimen for patients with pT2N3cM0 Stage IIIC breast cancer, which included epirubicin and cyclophosphamide (EC) (100 mg/m^2^ and 600 mg/m^2^, respectively) four times every 3 weeks, followed by docetaxel (DTX) (75 mg/m^2^) and trastuzumab (T-mab) (8 mg/kg loading dose and 6 mg/kg thereafter) four times every 3 weeks for adjuvant therapy. She received 70% of the full-dose of EC, 80% of the full-dose of DTX, and the full-dose of T-mab.

**Fig. 3:**
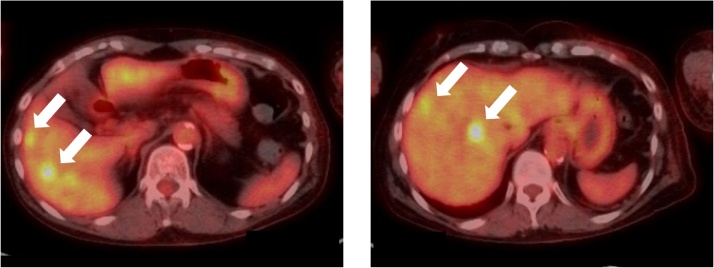
Positron emission tomography (PET)/computed tomography indicated multiple hot spots in the liver (arrows).

We used the vascular access (VA) for HD on the patient’s right forearm for the administration of anticancer drugs, which was on the opposite side of the surgical site. However, thrombosis developed in the VA after two rounds of EC. The patient also developed febrile neutropenia (FN), hemorrhagic cystitis, and anemia, which required thrombolytic therapy, antibiotics, granulocyte colony-stimulating factor, and blood transfusions. EC therapy was discontinued, and a central venous access port (CV port) was created at the right internal jugular vein. After that, the patient received four cycles of DTX and T-mab, followed by PMRT of 50 Gy in 25 fractions and 10 Gy in 5 fractions to the supraclavicular and internal mammary nodes. The patient then received full-dose T-mab monotherapy every 3 weeks, which was scheduled for 14 cycles and full-dose letrozole, which was scheduled for 10 years, as additional adjuvant therapy. However, one year after surgery, PET/CT indicated multiple metastases (Fig. 3), and blood tests revealed an increase in serum CA15-3.

Trastuzumab emtansine (T-DM1) was administered as the first-line treatment for the recurrence. She received a full-dose of T-DM1 (3.6 mg/kg every 3 weeks) for a total of 10 cycles among 32 weeks. However, the liver metastases did not respond, so she was administered for nine cycles of full-dose T-mab, pertuzumab (P-mab) (840 mg loading dose and 420 mg thereafter), and 70% of the full-dose of eribulin (1.4 mg/m^2^ on days 1 and 8 every 3 weeks) among 33 weeks. However, the liver metastases continued to grow, and we administered third-line treatment of full-dose T-mab, P-mab, and weekly paclitaxel (PTX) at 70% of the full dose (90 mg/m^2^) among 29 weeks. The third-line treatment was administered for nine cycles, and a partial response was observed in the liver metastases. However, the patient refused to continue the therapy because of grade 2 systemic edema and numbness in the fingers. Currently, full-dose T-mab, P-mab, and 60% of the full dose of vinorelbine (VNR; 25 mg/m^2^ on days 1 and 8 every 3 weeks) are being administered as the fourth-line treatment. She has survived more than 25 months after the initial detection of recurrence of breast cancer and maintained quality of life.

## 3 Discussion

In this report, we describe a patient with metastatic breast cancer who was undergoing HD. She had favorable survival and acceptable quality of life using a reduced dosage and several types of drugs, despite the difficulty of dose control. Drug dosages may need to be adjusted in some patients, but the lack of appropriate drug dosage can reduce overall survival [5]. A previous study reported that patients receiving a higher relative dose intensity (RDI) of anticancer drugs had better clinical outcomes than those receiving a lower RDI among patients with metastatic solid tumors, including breast cancer [6], but there are currently no data in patients undergoing HD.

The survival of patients with breast cancer who are receiving dialysis is still unknown. CKD is not a significant factor in overall survival or disease-free survival in patients with breast cancer [7]. In fact, the patient in the present report had a favorable survival even after breast cancer recurrence. However, the number of cases in the literature is limited, and there is no other report describing the survival of patients with breast cancer who are receiving HD.

Regarding the treatment of breast cancer patients who are receiving HD, the most important clinical issue is drug selection and dosage adjustment. The recommendations for dosage adjustment for oncological therapy among patients receiving HD are shown in Table 1 [8,9]. Most drugs are safe to administer in patients receiving HD, and methotrexate is the only drug that should be avoided. It is recommended that capecitabine, cyclophosphamide, DTX, doxorubicin, epirubicin, and VNR be administered at reduced doses. There are insufficient data on HD patients receiving tamoxifen, anastrozole, letrozole, and newer targeted agents, such as cyclin-dependent kinase 4/6 inhibitors, lapatinib, T-mab, P-mab, T-DM1, and atezolizumab. However, even drugs that do not require dose reduction should be reduced if they cause myelotoxicity or cardiotoxicity. However, these recommendations are general; therefore, for patients without CKD, dose reduction depends on the patient’s adverse events (AEs). In this case, we reduced the dose of EC to 70%, that of DTX to 80%, that of eribulin to 70%, that of PTX to 70%, and that of VNR to 60%. Even drugs that are generally administered at the full dose had to be reduced due to AEs, including thrombosis, FN, hemorrhagic cystitis, and anemia. Although there is no recommended dosage reduction in the literature for T-DM1, T-mab, P-mab, and letrozole [8], we were able to administer these drugs at the full dose without any major AEs.

**Table 1:** Summary of pharmacokinetics and safety of oncological therapies for breast cancer in hemodialysis patients.

Drug	Elimination	Dose reduction in HD Bednarek A8)	Recommended dose in HD Janus N9)	safety
capecitabine	Urinary	Recommended dose reduction by 50%	No data	Safe (limited data)
cyclophosphamide	Urinary	Recommended dose reduction by 20%	Reduction of 25%	Hemorrhagic cystitis
docetaxel	Faeces	Not specified	65 mg/m^2^	Safe
paclitaxel	Faeces	Not specified	Standard dose	Safe
doxorubicin	Faeces	Recommended dose reduction by 20%	Standard dose	Inceased risk of cardiotoxicity
epirubicin	Faeces	Recommended dose reduction	Standard dose	Inceased risk of cardiotoxicity
vinorelbine	Faeces	Probably necessary (up to 50%)	Reduction of 20%–33%	Inceased myelotoxicity (limited data)
tamoxifen	Faeces	Not indicated	−	Safe
anastrozole	Faeces	Not indicated	−	Safe
letrozole	Urinary	Not specified	−	No data
palbociclib	Faeces	Not specified	−	No data
lapatinib	Faeces	Not indicated	−	Safe (limited data)
trastuzumab	No data	Not indicated	−	Inceased risk of cardiotoxicity
pertuzumab	No data	Not indicated	−	No data
trastuzumab emtansine	Faeces	Not indicated	−	Inceased risk of cardiotoxicity
atezolizumab	No data	Not indicated	−	Safe (very limited data)

Another issue is among patients undergoing HD is the VA, particularly for patients who undergo axillary dissection. If the dissection range includes the VA site, it may be preferable to place a VA on the opposite side of the surgical site prior to operation to reduce the risk of VA issues. Another alternative is to use a temporary VA for the short term. However, no previous report was found. It might be useful to confirm the effect of the operation on the original VA. In our case, the patient’s VA was on the opposite side of the surgical site, so it was unaffected by the surgery.

Intravenous administered of anticancer drugs should be considered. In our case, the venous pressure in the internal jugular vein was increased by the VA on the non-affected side. It is usually recommended to create a CV port to prevent vasculitis caused by anticancer drugs, but we need to consider the risks of venous pressure increase. We initially decided to administer anticancer drugs from the VA for HD, but the patient developed thrombosis, and we created a CV port via the internal jugular vein on the VA side without any complications. This case is reported in line with the SCARE Guideline [10].

## 4 Conclusion

We report a case of breast cancer diagnosed during HD with multiple liver metastases that developed during postoperative adjuvant therapy. It is very difficult to manage patients undergoing HD with the appropriate drugs and dosage to improve survival and quality of life.

## Declaration of Competing Interest

None.

## Funding

None.

## Ethical approval

None.

## Consent

Written informed consent was obtained from the patient for publication of this case report and accompanying images. A copy of the written consent is available for review by the Editor-in-Chief of this journal on request.

## Author contribution

Megumi Matsumoto conceptualized the study. Hiroshi Yano, Ryota Otsubo, and Aya Tanaka collaborated in the patient’s care. Takeshi Nagayasu provided input on the manuscript.

## Registration of research studies

Researchregistry 6411.

## Guarantor

Megumi Matsumoto.

## Provenance and peer review

Not commissioned, externally peer-reviewed.
